# Pan-Cancer Characterization Identifies SLC19A1 as an Unfavorable Prognostic Marker and Associates It with Tumor Infiltration Features

**DOI:** 10.3390/biomedicines13030571

**Published:** 2025-02-25

**Authors:** Yimin Pan, Zhichen Liu, Changwu Wu

**Affiliations:** 1 Department of Neurosurgery, Xiangya Hospital, Central South University, No. 87 Xiangya Road, Changsha 410008, China; 2 Department of Orthopedics, Xiangya Hospital, Central South University, No. 87 Xiangya Road, Changsha 410008, China; 3 National Clinical Research Center for Geriatric Disorders, Xiangya Hospital, Central South University, Changsha 410008, China

**Keywords:** pan-cancer, SLC19A1, prognostic marker, TCGA, immune infiltration

## Abstract

**Background:** Recent studies have identified solute carrier family 19 member 1 (SLC19A1) as a second messenger transporter that regulates massive immune-related signaling cascades, but current studies provide limited information. This study aims to evaluate its role and the potential mechanisms across various cancers. **Methods:** We analyzed multi-omics data from a pan-cancer cohort to evaluate SLC19A1 expression and its association with multiple features, including prognosis, tumor stemness, genome instability, and immune infiltration. Immunofluorescence staining was performed to validate SLC19A1 expression in tumor tissues and its relationship M2 macrophages. In addition, we used web tools such as ROCplotter to evaluate the association between SLC19A1 and response to chemotherapy and immunotherapy. **Results:** SLC19A1 was found to be overexpressed in multiple cancer types compared to normal tissues, correlating with poor prognosis. High SLC19A1 levels were associated with increased genomic instability and immune suppression. In addition, SLC19A1 was negatively correlated with CD8+ T-cell infiltration and positively correlated with M2 macrophage infiltration. The association of SLC19A1 with M2 macrophages was confirmed in multiple immunofluorescence staining. Finally, SLC19A1 was associated with the response to chemotherapy and immunotherapy in a variety of tumors. **Conclusions:** Our findings position SLC19A1 as a novel unfavorable prognostic marker in cancer, closely linked to immune suppression and genomic instability. This study highlights the need for further exploration of SLC19A1 as a therapeutic target and its implications in cancer treatment strategies.

## 1. Introduction

Solute carrier family 19 member 1 (SLC19A1) functions as a bi-directional folate transporter that imports folate and its structural analogs anti-folates while exporting organic anions, including thiamine derivatives, nucleotides, and inorganic anions [1,2]. Deletion of the downstream protein encoded by SLC19A1 has been shown to cause folate deficiency in a murine model, resulting in premature death of embryos due to severe hematopoietic dysfunction [3]. Clinically, mutations leading to its deletion or dysfunction are associated with various diseases caused by folate deficiency, including anemia, cardiovascular diseases, etc. [4,5]. Functional expression of SLC19A1 also correlates with sensitivity to several anti-folate chemotherapeutic agents, including methotrexate and pemetrexed, due to the critical role of its encoded protein in the transport of structurally similar anti-folates [4,6,7,8,9].

In addition to its function as a folate transporter, more recently, it has also been reported to transport cyclic dinucleotides in different cells [10,11]. Cyclic dinucleotides are widely distributed in various cells and act as an important signaling molecule [12,13]. Not only the cyclic dinucleotide 2′3′-cGAMP produced by human cells but also the analogs produced by bacteria can act as a second messenger to activate the stimulator of interferon genes (STING) pathway, thereby initiating massive downstream signaling cascades [14,15,16,17,18,19,20,21,22,23,24,25,26,27,28,29]. The intracellular transport of these cyclic dinucleotides mediated by SLC19A1 ensures the downstream signaling pathways, as these second molecules must enter cells to function [10,11]. Therefore, the importance of cyclic dinucleotides in anti-cancer and anti-infective immunity highlights the critical role of their intracellular transporter SLC19A1 in infection and cancer immunology [29,30,31,32,33].

The rapid and infinite proliferation of cancer cells is maintained by a vigorous metabolism that requires increased transport of folate for the biosynthesis of macromolecules. Several chemotherapeutic agents have been invented based on their similar structure to folate, which could replace folate to be imported by metabolically active tumor cells, thus interfering with folate-dependent biosynthesis [1,34,35,36]. Given the critical role of cyclic dinucleotide 2′3′-cGAMP in cancer immunity, SLC19A1 may serve as a potential target, as it has been shown to be the exchanger for both folate and 2′3′-cGAMP in mammalian cells [4,10,11,37,38]. Although studies have reported its association with osteosarcoma and leukemia resistance to chemotherapies, the possible mechanisms of SLC19A1 in most of the other tumors have not yet been elucidated [7,39,40]. Interestingly, a prospective pharmacogenetic study found that solute carriers, including SLCO1B1 and SLC19A1, were associated with chemotherapy response in metastatic colorectal cancer patients [41]. Similar findings have also been reported in breast and lung cancers [42,43,44]. Specifically, SLC19A1 was identified as a predictor of complete response to neoadjuvant chemotherapy in hormone-sensitive breast cancer [44]. In pemetrexed-treated non-small cell lung cancer, patients with homozygous variant genotypes had a more favorable survival [43]. It was also reported as a biomarker for multiple myeloma [45]. These encouraging findings have inspired us to consider the value of SLC19A1 in risk stratification and bedside decision making for cancer patients. It was hypothesized that SLC19A1 plays a vital role in tumorigenicity and may serve as a potent breakthrough for the activation of innate immunity to kill cancer cells. Considering pan-cancer characterization and multi-omics analysis can provide initial insights for potential mechanisms [46,47], multi-omics data were analyzed on a pan-cancer scale using algorithms including deconvolution to gain a comprehensive understanding of SLC19A1 in cancer biology. Specifically, we present the first comprehensive pan-cancer analysis of SLC19A1, leveraging transcriptomic, proteomic, and epigenomic datasets from 33 cancer types. Unlike previous studies, our work uniquely integrated genomic instability metrics, tumor stemness indices, and immune infiltration features to unravel the multifaceted roles of SLC19A1. Notably, we uncovered its previously unrecognized association with M2 macrophage polarization and CD8+ T-cell exclusion, providing mechanistic insights into immune evasion. Furthermore, we established SLC19A1 as a predictor of chemotherapy and immunotherapy resistance, a finding with direct translational relevance. These analyses address critical gaps in understanding how SLC19A1 orchestrates tumor progression and immune suppression, positioning it as a novel therapeutic target for precision oncology.

## 2. Methods

### 2.1. Transcriptomic and Proteomic Difference Between Tumor and Adjacent Normal Tissue

The abbreviations for cancers are shown in Table 1. RNA expression level of primary tumor tissues and matched adjacent normal tissues were compared and visualized with scattered bar charts using TIMER2.0 (http://timer.cistrome.org/) (accessed on 1 June 2023) with the function “Gene_DE” in the “Cancer Exploration” module [48,49,50]. As some cancer cohorts (ACC, DLBC, HNSC, LAML, LGG, MESO, OV, SARC, SKCM, TGCT, THYM, UCS, and UVM) lack paired normal tissue, transcriptomic data of 54 normal tissues in the Genotype-Tissue Expression (GTEx) database were included for the differentially comparison (https://www.gtexportal.org/) (accessed on 1 June 2023). We downloaded the uniformly normalized TCGA Pan-Cancer dataset from the TCGA PanCan Atlas project in the UCSC database (https://xenabrowser.net/) (accessed on 1 June 2023). This dataset utilized the EB++ algorithm to eliminate batch effects and was normalized using the upper quartile of batch-corrected RSEM data.

The mRNA expression level of SLC19A1 in different pathological stages of specific cancers in the TCGA cohorts was visualized using the function “Stage Plot” in the module “Expression Analysis” of Gene Expression Profiling Interactive Analysis (GEPIA2, http://gepia2.cancer-pku.cn/) (accessed on 5 June 2023) [51], and “log2(TPM + 1)” was used for the normalization by the platform. *p* < 0.05 was used as the threshold for differential expression analysis.

Proteomic data of different solid tumors and their paired normal tissues from Clinical Proteomic Tumor Analysis Consortium (CPTAC) and the International Cancer Proteogenome Consortium (ICPC) datasets were compared and visualized with bar charts using the University of Alabama at Birmingham Cancer data analysis Portal (UALCAN, http://ualcan.path.uab.edu/analysis-prot.html) (accessed on 5 June 2023) [52,53,54,55]. Protein data were normalized by UALCAN. In brief, protein expression values downloaded from the CPTAC data portal were log2 normalized in each sample. Then, a Z-value for each sample for each protein was calculated as standard deviations from the median across samples. The expression of SLC19A1 encoding protein between tumor and normal tissues in the breast cancer, glioblastoma, lung squamous cell carcinoma, live hepatocellular carcinoma, uterine corpus endometrial carcinoma, and lung adenocarcinoma were visualized and selected, of which the *p*-values are all less than 0.01.

### 2.2. Clinical Association and Prognostic Value of SLC19A1

For Kaplan–Meier survival analysis based on mRNA expression, transcriptomic and matched follow-up data of SLC19A1 were obtained from the TCGA pan-cancer cohorts and visualized using the R package “survival” with the function “survfit” [56]. The high- and low-expression groups were divided according to the median value of the mRNA expression level of SLC19A1. Additionally, the matched overall survival (OS), disease-specific survival (DSS), and progression-free interval (PFI) of each patient were also obtained. The R package “survival” was also used to calculate the hazard ratio of SLC19A1 in each cancer cohort and to visualize the results using the “coxph” function.

For Kaplan–Meier survival analysis based on copy number variation (CNV), the Tumor Immune Dysfunction and Exclusion (TIDE) platform was used to analyze and visualize the association between CNV and OS using the “Query Gene” module and the “Copy_Number” function (http://tide.dfci.harvard.edu/query/) (accessed on 5 June 2023) [57,58]. CNV data were standardized by the TIDE platform. According to previous studies [47,59], deep deletions in CNV data are represented by −2, shallow deletions by −1, diploidy by 0, low-level amplifications by 1, and high-level amplifications by 2, and the data were normalized using z-score.

For Kaplan–Meier survival analysis based on the epigenetic modification, the TIDE platform was also used to analyze and visualize the association between the DNA methylation level of SLC19A1 and OS using the module “Query Gene” and the “Methylation” function [57,58]. Methylation data were normalized by the TIDE platform. For genes with multiple probes, only the methylation data of the probe with the strongest negative correlation between methylation signal and gene expression was included, and the data were subsequently normalized using z-score.

### 2.3. Visualization of Copy Number Alterations (CNAs) Across TCGA Pan-Cancer Cohorts

To visualize the CNAs of SLC19A1 on a pan-cancer scale, the cBioPortal platform was used to integrate the mutation data from the TCGA pan-cancer cohorts (https://www.cbioportal.org/) (accessed on 10 June 2023) [60,61,62]. In the “TCGA Pan-Cancer Atlas” query, datasets were selected using the “Query by Gene” function, and SLC19A1 was then entered for the integrated analysis. In the Cancer Type Summary module, alterations across different cancer types were visualized. In the Mutations module, the major mutated sites of SLC19A1 DNA were also visualized.

### 2.4. Correlation Between Genomic Heterogeneity and Gene Expression

Genomic heterogeneity including tumor mutational burden (TMB), microsatellite instability (MSI), and neoantigen were annotated, and their association with the expression of SLC19A1 in each cancer cohort was visualized using the R package “maftools” [63]. The Level 4 Simple Nucleotide Variation dataset for all TCGA samples, processed by the MuTect2 software (version v4.1.0.0), was downloaded from the GDC (https://portal.gdc.cancer.gov/) (accessed on 10 June 2023). Then the TMB for each tumor was calculated using the “tmb” function of the R package “maftools”. The MSI data and neoantigen data were obtained from the previously published studies [64,65]. MSI analysis with MANTIS was performed as previously described [66]. Potential neoantigenic peptides were identified using NetMHCpan v3.0 [67], based on HLA types derived from RNA-seq using OptiType [68].

In addition, the association between homologous recombination deficiency (HRD), ploidy, tumor purity, cancer stemness, and the expression of SLC19A1 was analyzed and visualized using the Sangebox platform (http://sangerbox.com/) (accessed on 10 June 2023) [69]. The differentially methylated probes-based stemness index (DMPsi) was obtained from a published study [70], DMPsi was generated based on three signatures, and the specific details can be found in the source literature. After integrating the TMB, MSI, neoantigens, DMPsi, and gene expression data of SLC19A1 of the samples, the correlation between SLC19A1 and these indicators was calculated using Spearman correlation analysis.

### 2.5. Correlations with Epigenetic Modifications

Using the “Methylation” function as mentioned above in the TIDE platform, the correlation between the promoter methylation level of SLC19A1 and cytotoxic T lymphocytes (CTLs) was visualized in scatter plots. Their associations with patient survival in different cancers were also depicted in Kaplan–Meier curves at the same time as mentioned in this module.

Regulators of RNA epigenetic modifications, including N1-methyladenosine (m1A), 5-methylcytosine (m5C), and N6-methyladenosine (m6A), were obtained from previously published studies [71,72,73]. To assess the potential regulation of SLC19A1 in these modifications, their correlations were calculated and presented in a heatmap using Sangerbox platform [69].

### 2.6. Identification of Correlated Genes and Gene Enrichment Annotations

To explore the potential interacted proteins with SLC19A1, the STRING database (https://string-db.org/) (accessed on 10 June 2023) was used to build the protein–protein association network based on their systemic collection of published data [74]. The GEPIA 2.0 platform was used to identify genes that expressed correlatively with SLC19A1 based on the transcriptomic data of TCGA pan-cancer cohorts [51]. Five genes with largest correlation coefficients were selected and visualized by the TIMER 2.0 platform and GEPIA 2.0 platform [48,49,50,51], and 100 genes were used for the KEGG pathway and GO enrichment annotations by the R package “clusterProfiler” [75]. Gene Set Enrichment Analysis (GSEA) was performed by the GSEA software (http://software.broadinstitute.org/gsea/index.jsp) (accessed on 10 June 2023), and the referenced hallmark gene set for the annotation was obtained from the Molecular Signature Database (http://software.broadinstitute.org/gsea/index.jsp) (accessed on 10 June 2023) [76,77].

### 2.7. Prediction of Tumor Infiltrated Cells and the Association with Immune Checkpoints

The R package “ESTIMATE” was used to assess tumor purity, infiltrated stromal cells, and immune cells based on the transcriptomic data of the TCGA pan-cancer cohorts by calculating the ESTIMATE score, stromal score, and immune score, respectively [78]. Bar charts were used to visualize the correlation between SLC19A1 expression and the ESTIMATE score, stromal score, and immune score across different cancer types in the TCGA cohorts, while scatter plots were used to show the correlation between SLC19A1 expression and these scores in the most correlated tumors.

Immune checkpoints were obtained from a published study [65], and their correlation coefficients and *p*-values with the SLC19A1 expression in different cancers were calculated and visualized by a heatmap by the Sangebox platform (http://sangerbox.com/) (accessed on 20 June 2023) [69]. The correlation between SLC19A1 expression and the expression of chemokines, receptors, and MHC molecules was visualized simultaneously. The TISIDB (http://cis.hku.hk/TISIDB/) (accessed on 20 June 2023) was used to assess the differential expression of SLC19A1 between previously defined immune subtypes across multiple cancer types [79].

For the estimation of infiltrated immune cells, TIMER2.0 was applied to the transcriptomic data of the TCGA pan-cancer cohorts based on deconvolution algorisms [48,49]. Spearman correlation coefficients were calculated and applied to assess the correlation between the mRNA expression of SLC19A1 and predicted infiltration level of immune cells.

### 2.8. Immunofluorescence Staining

Tumor tissues and corresponding normal tissues were obtained from Xiangya Hospital of Central South University. The collection of tissues was approved by the Medical Ethics Committee of Xiangya Hospital of Central South University (Approval number: 202303046), and written informed consent was provided by all of the patients. The tumor tissue and normal tissue used in this study were stained by the rabbit anti-SLC19A1 antibody (Proteintech, Rosemont, IL, USA, Cat No. 25958-1-AP) diluted at 1:300, and the panel for M2 macrophages, including mouse anti-CD68 antibody (Cat No. ab955; Abcam, Cambridge, UK), was diluted at 1:300 and rat anti-CD163 antibody (Cat No. ab289979; Abcam, Cambridge, UK) diluted at 1:300. The antigen retrieval was initially performed for these tumor and normal tissue slices. These slices were then deparaffinized and blocked using 3% H_2_O_2_ and 2% bovine serum albumin. The above-mentioned antibodies were diluted accordingly and were subsequently applied for the incubation of the slices. After the incubation with secondary antibodies including Goat Anti-Rabbit IgG H&L (Alexa Fluor^®^ 488, Cat No. ab150077; Abcam, Cambridge, UK) diluted at 1:1000, Goat Anti-Mouse IgG H&L (Alexa Fluor^®^ 555, Cat No. ab150118; Abcam, Cambridge, UK) pre-adsorbed diluted at 1:1000, and Goat Anti-Rat IgG H&L (Alexa Fluor^®^ 594, Cat No. ab150160; Abcam, Cambridge, UK) diluted at 1:1000, a counterstaining of 4′,6-diamidino-2-phenylindole (DAPI) was performed to mark the nucleuses. Quantification of immunofluorescence staining was performed using ImageJ software (version 1.54m). Three views of each tumor were taken for cell counting. Images in the blue channel (DAPI) were used to quantify total cell number, while the green, red, and yellow channels were used to quantify SLC19A1-positive, CD206-positive, and CD68-positive cells, respectively. Cells labeled in the green channel were considered to be SLC19A1-positive. Corresponding cells in the red (CD206) and yellow (CD68) channels were labeled as macrophages. The percentage of positive cells (positive cells/DAPI-positive cells) was therefore used to compare SLC19A1-positive cells and macrophages between different groups. Student’s *t*-test was used to determine whether SLC19A1 or macrophage infiltration was different between tumor and normal samples.

### 2.9. RNA-Seq of Glioma Samples

Additionally, 151 glioma samples were obtained from Xiangya Hospital of Central South University. The collection of tissues was approved by the Medical Ethics Committee of Xiangya Hospital of Central South University (Approval number: 202401003), and written informed consent was provided by all of the patients. RNA-seq was performed as described previously [80]. Briefly, total RNA was extracted from the tissue samples using the TRIzol^®^ reagent as per the manufacturer’s instructions. Sample quality and quantity were assessed using the Agilent 5300 Bioanalyzer and ND-2000 NanoDrop, respectively. Library preparation and sequencing were conducted at Shanghai Majorbio Bio-pharm Biotechnology, following Illumina’s guidelines. Next, 1 μg of total RNA was used to construct the RNA-seq transcriptome library via the Illumina^®^ (San Diego, CA, USA) Stranded mRNA Prep, Ligation kit. This involved polyA-based mRNA isolation, fragmentation, and double-stranded cDNA synthesis using random hexamers. The cDNA underwent end-repair, PCR amplification, and quantification before paired-end sequencing on the NovaSeq 6000 platform. Raw sequencing reads were trimmed and quality-filtered using fastp. The cleaned reads were then aligned to the reference genome with HISAT2. Read assembly was performed using StringTie, and gene expression levels were quantified using RSEM (version v1.3.3). Finally, the RNA-seq data from 151 glioma samples were transformed into transcript per million.

## 3. Results

### 3.1. SLC19A1 Is Commonly Dysregulated at the Transcriptomic Level and Serves as a Risk Factor

**Figure 1:**
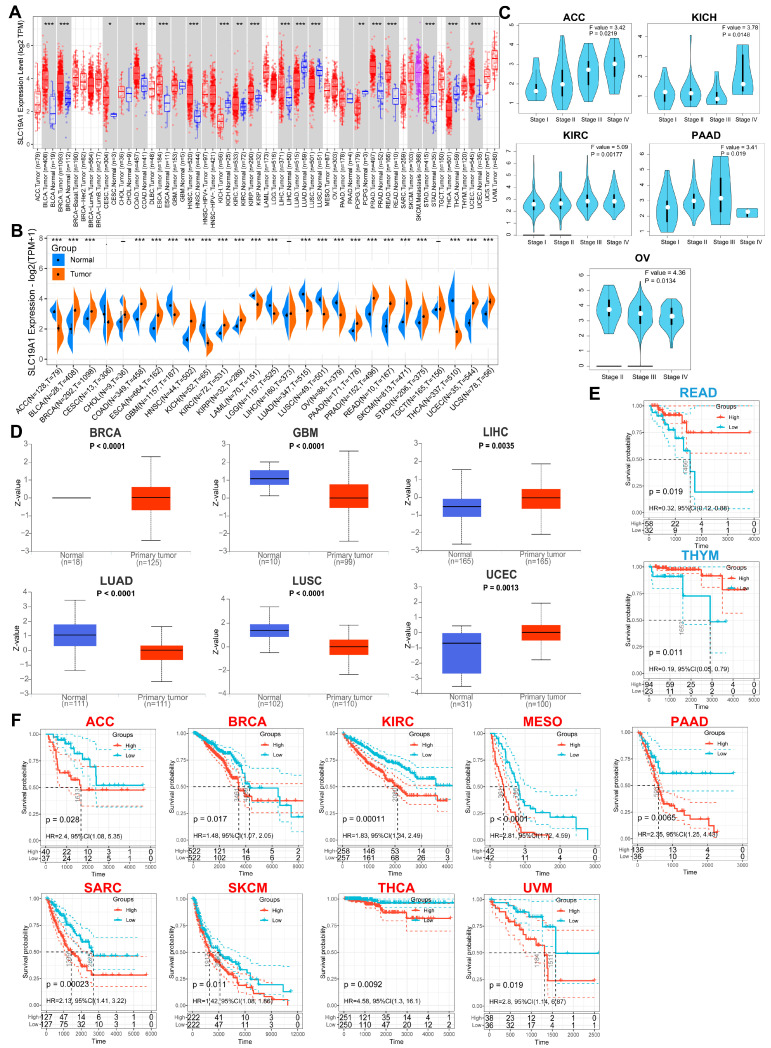
**SLC19A1 is differentially expressed and predicts the survival of cancers.** (**A**) Transcriptomic expression level of SLC19A1 in tumor and normal tissues in the TCGA pan-cancer cohorts. (**B**) Transcriptomic expression level of SLC19A1 in tumor and normal tissues in the TCGA pan-cancer cohorts integrating data from 54 normal tissues from the GTEx database. (**C**) Transcriptomic expression level of SLC19A1 at different pathological stages in ACC, KICH, KIRC, PAAD, and OV. (**D**) Proteomic expression level of SLC19A1 in BRCA, GBM, LUSC, LIHC, UCEC, and LUAD and their adjacent normal tissues. (**E**) Kaplan–Meier curves of patients with high and low transcriptomic expression level of SLC19A1 in READ and THYM cohorts. (**F**) Kaplan–Meier curves of patients with high and low transcriptomic expression level of SLC19A1 in ACC, BRCA, KIRC, MESO, PAAD, SARC, SKCM, THCA, and UVM. * *p* < 0.05, ** *p* < 0.01, and *** *p* < 0.001.

For an overview of the transcriptomic expression of SLC19A1 on a pan-cancer scale, the mRNA expression profile of TCGA pan-cancer cohorts was obtained and visualized. This demonstrated that SLC19A1 has a broad differential expression across these cancers (Figure 1A,B). The mRNA expression of SLC19A1 was significantly higher than normal tissue in 14 types of cancers, including BLCA, BRCA, COAD, ESCA, HNSC, KIRC, KIRP, PAAD, PRAD, READ, SKCM, STAD, UCES, and UCS, while the opposite was shown in 9 other types of cancers, including ACC, GBM, KICH, LAML, LGG, LUAD, LUSC, OV, and THCA (Figure 1B). Only four types of cancers showed no differential expression of SLC19A1 across the twenty-seven types of cancer studied (Figure 1B). The clinical status of these cancers was also analyzed, indicating that the mRNA of SLC19A1 is expressed differentially across different clinical stages in ACC, KICH, KIRC, PAAD, and OV (Figure 1C). At the protein level, the expression of SLC19A1 in cancers and normal tissue was compared, showing that the protein encoded by SLC19A1 is expressed differentially between six types of cancers, including BRCA, GBM, LUSC, LIHC, UCEC, and LUAD (Figure 1D). The follow-up survival data suggest that patients with a higher expression of SLC19A1 have a better survival in READ and THYM (Figure 1E). However, SLC19A1 was identified to more commonly serve as a risk factor in nine cancers, including ACC, BRCA, KIRC, MESO, PAAD, SARC, SKCM, THCA, and UVM (Figure 1F). Univariate Cox analysis of OS, DSS, and PFI indicated that SLC19A1 is more frequently associated with an increased risk across different cancers (Supplementary Figure S1). Briefly, SLC19A1 was widely dysregulated at the transcriptomic level in different cancers and commonly associated with poor prognosis on a pan-cancer scale. Further, we conducted RNA-seq on tumor samples collected from 151 glioma patients to construct an independent clinical cohort for complementary analysis. We found that compared to grade I–II gliomas, grade III–IV gliomas exhibited relatively higher SLC19A1 expression (Supplementary Figure S2A), suggesting a potential role of SLC19A1 in promoting the malignant progression of gliomas. Further prognostic analysis revealed that patients with high SLC19A1 expression had significantly poorer outcomes among all glioma samples (Supplementary Figure S2B). Upon stratification, we observed that patients with high SLC19A1 expression had poorer prognosis in the GBM group (Supplementary Figure S2C), whereas there was no significant difference between the high and low SLC19A1 expression groups in LGG patients (Supplementary Figure S2D).

### 3.2. Genetic Alteration of SLC19A1 Is Associated with Neoplastic Biomarkers

**Figure 2:**
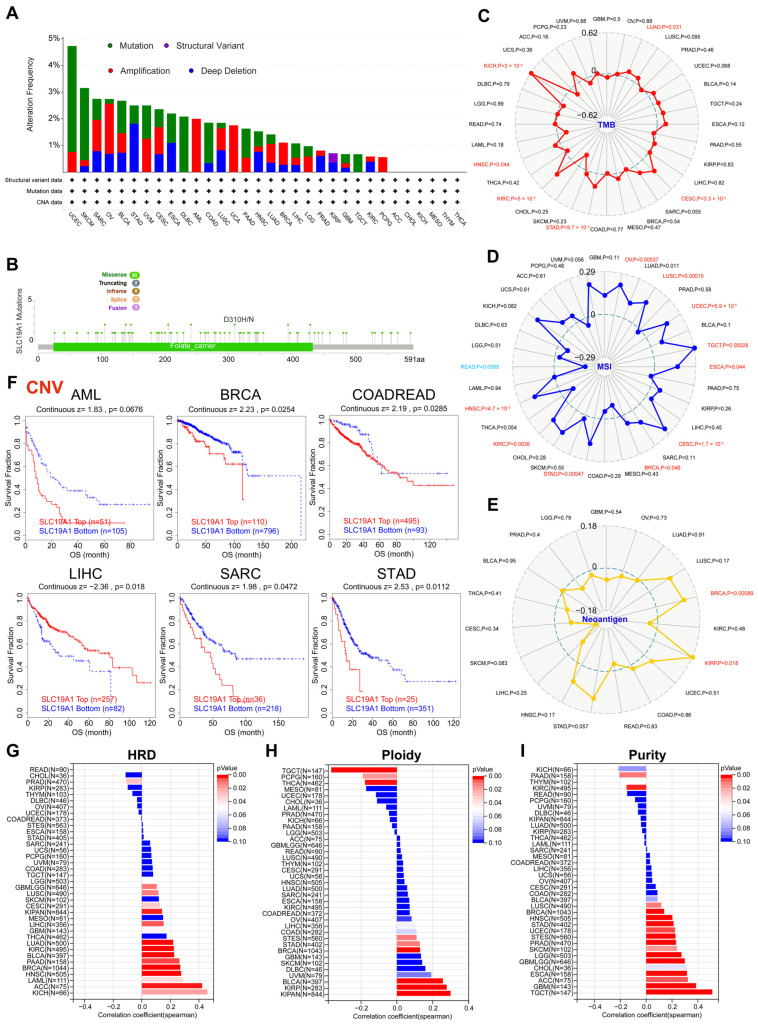
**SLC19A1 is associated with genomic instability in cancers.** (**A**) Genetic alterations of cancers in the TCGA pan-cancer cohorts. (**B**) A summary of the most frequently occurring types and sites of SLC19A1 mutations. (**C**) The radar plot demonstrates the correlation between SLC19A1 expression and the predicted level of TMB. A greater degree of correlation between the two variables results in a greater distance between the point representing the tumor and the center. (**D**) The radar plot illustrates the correlation between SLC19A1 and MSI. (**E**) A similar radar plot is presented here, but it illustrates the correlation between SLC19A1 and neoantigen. (**F**) Kaplan–Meier curves of patients exhibiting high and low copy number alterations (CNAs) of SLC19A1 in acute myeloid leukemia (AML), breast invasive carcinoma (BRCA), colon adenocarcinoma (COAD), rectum adenocarcinoma (READ), liposarcoma (LIHC), and stomach adenocarcinoma (STAD). (**G**) The bar chart demonstrates the correlation between SLC19A1 expression and the predicted level of homologous recombination deficiency (HRD). The distance from the center to both sides of the plot indicates the correlation coefficient, while the color of the bars indicates the *p*-values. (**H**) The bar chart illustrates the correlation between SLC19A1 and ploidy. (**I**) The bar chart shows the correlation between SLC19A1 and tumor purity.

Considering the dysregulation of SLC19A1 at the transcriptomic and proteomic levels, the genetic alteration profile was summarized on a pan-cancer scale (Figure 2A). The majority of cancers (*n* = 26/32) included in the TCGA pan-cancer cohorts were identified to exhibit at least one type of genetic alteration (Figure 2A). UCEC demonstrated the highest alteration frequency of approximate 5%, while ACC, CHOL, KICH, THYM, and THCA exhibited no alteration (Figure 2A). Among all types of alterations in different cancers, missense mutations were the dominant type (n = 83/86), most of which were located in sites that act as folate carriers (Figure 2B). The expression of SLC19A1 was identified to be significantly correlated with TMB status in KICH, HNSC, KIRC, STAD, CESC, and LUAD, while a significant correlation between SLC19A1 and MSI was identified in READ, HNSC, KIRC, STAD, BRCA, CESC, ESCA, TGCT, UCEC, LUSC, and OV (Figure 2C,D). However, neoantigen was investigated but only found to correlate with SLC19A1 in BRCA and KIRP (Figure 2E). Notably, most of these correlations were found to be positive, except for READ (Figure 2C–E). The expression of SLC19A1 was also identified to be commonly correlated positively with HRD and tumor purity (Figure 2G,I). But for ploidy, SLC19A1 correlated positively in KIPAN, KIRP, UVM, BRCA, STAD, and STES and negatively in TGCT, PCPG, and THCA (Figure 2H). The Kaplan–Meier curves suggest a negative association between SLC19A1 alteration and survival in AML, BRCA, and COAD and READ, SARC, and STAD and a positive association in LIHC (Figure 2F). These findings suggest SLC19A1 is commonly mutated in the majority of cancers. Consistent with the transcriptomic data, genetic alteration of SLC19A1 also suggests an unfavorable prognosis and is associated with several neoplastic biomarkers, including TMB, MSI, neoantigen, HRD, ploidy, and tumor purity.

### 3.3. SLC19A1 Is Associated with Cancer Stemness, Methylation, and RNA Regulatory Genes

**Figure 3:**
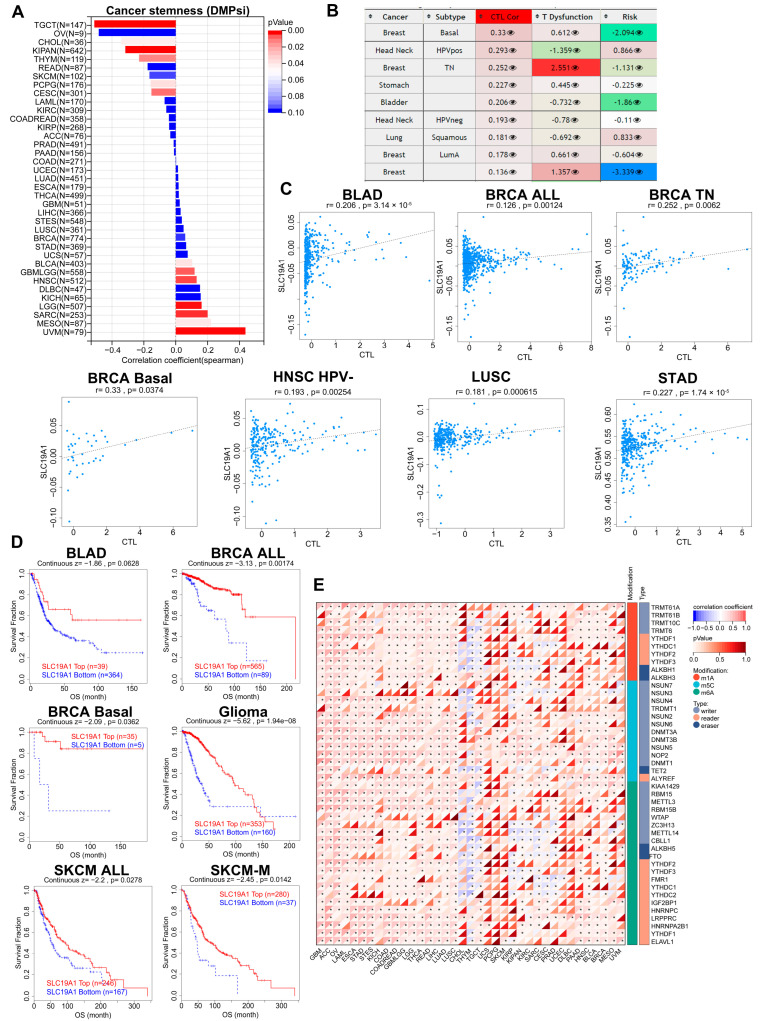
**SLC19A1 is involved in cancer stemness, methylation, and RNA regulatory genes.** (**A**) The bar chart demonstrates the correlation between SLC19A1 expression and cancer stemness (DMPsi). (**B**) The table presents the correlation between the methylation level of SLC19A1 and CTLs, T-cell dysfunction, and the risk of SLC19A1 methylation in survival analysis. (**C**) The scatterplots demonstrate the correlation between the methylation level of SLC19A1 and CTLs. (**D**) The Kaplan–Meier curves illustrate the survival of patients with a high and low methylation level of SLC19A1. (**E**) The heatmap demonstrates the correlation between the transcriptomic expression of SLC19A1 and regulators of RNA modifications. * *p* < 0.05.

In order to further explore the function of SLC19A1, the correlation between the expression of SLC19A1 and cancer stemness was analyzed (Figure 3A), demonstrating that the expression of SLC19A1 correlated positively with cancer stemness in UVM, MESO, SARC, LGG, HNSC, GBM, LGG, and BLCA but negatively in TGCT, CHOL, KIPAN, THYM, PCPG, and CESC (Figure 3A). Considering that cancer stemness is involved in oncogenic dedifferentiation via multiple pathways, including regulation of methylation status, SLC19A1 promoter methylation levels were examined and found to be significantly correlated with the abundance of cytotoxic T lymphocytes (CTLs) predicted in several cancer types (Figure 3B). Specifically, these cancers and subtypes are BLAD, basal-like BRCA, triple-negative BRCA, all subtypes of BRCA, HPV-negative HNSC, LUSC, and STAD (Figure 3C). The SLC19A1 promoter status in these cancers all correlated positively with the CTLs (Figure 3C). Kalan–Meier curves suggest that higher SLC19A1 promoter methylation level predicts a better prognosis in BLAD, BRCA, basal-like BRCA, glioma, SKCM, and SKCM metastasis (Figure 3D). The association between SLC19A1 and RNA modification regulators was also explored (Figure 3D). The expression of SLC19A1 correlated positively with the expression of m1A, m5C, and m6A regulators in GBM, ACC, OV, LAML, ESCA, STAD, STES, KICH, COAD, READ, LGG, THCA, LIHC, LUAD, and LUSC (Figure 3E). For the rest of cancers analyzed, SLC19A1 was associated with at least one type of regulators of these three RNA modifications (Figure 3E). Therefore, the expression of SLC19A1 is associated with the epigenetic modification status, and the methylation of its promoter predicts a better prognosis in several types of cancers.

### 3.4. SLC19A1 May Contribute to the Oncogenic Cascades and Immunosuppression

**Figure 4:**
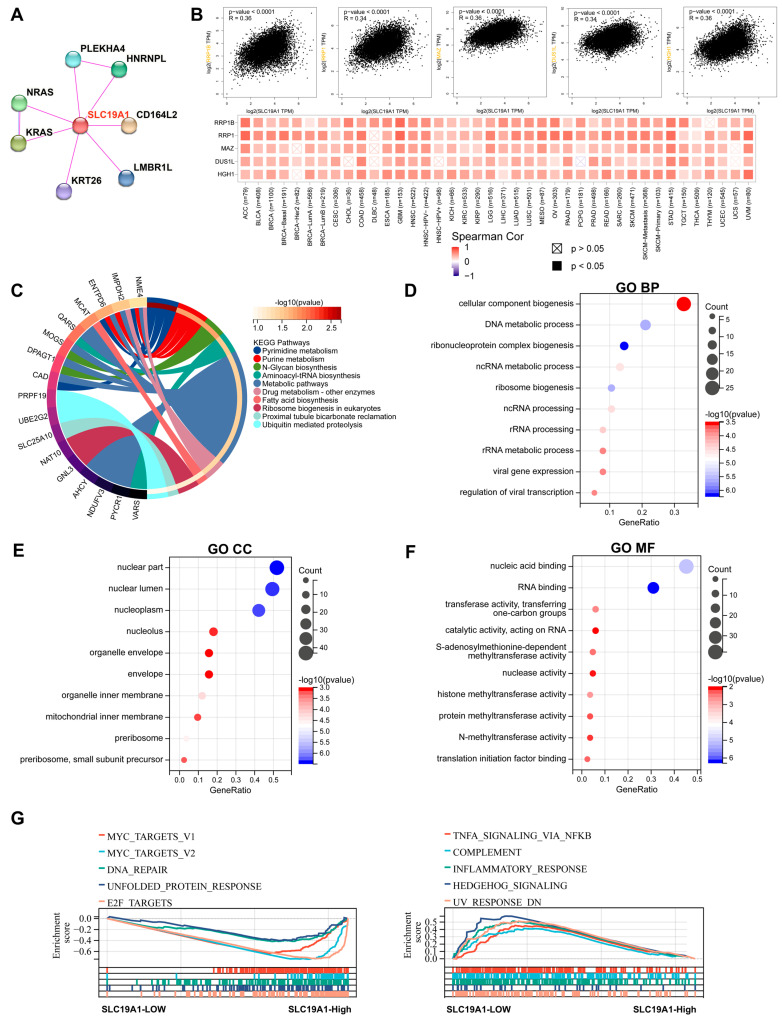
**SLC19A1 is involved in the oncogenic cascades and immunosuppression.** (**A**) The PPI network shows the interacted proteins with SLC19A1 according to the STRING database. (**B**) The scatterplots demonstrate the correlation between SLC19A1 expression and the expression of the genes with top five largest correlation coefficients. The heatmap below demonstrates the correlation between SLC19A1 and these genes in the TCGA pan-cancer cohorts. (**C**) The circular chart shows the KEGG annotation of genes that correlate with SLC19A1. The genes with the lowest *p*-values of each KEGG pathways are labeled. (**D**) The bubble plot shows the biological process (BP) of the GO annotation of these correlated genes. (**E**) The bubble plot shows the cellular component (CC) of the GO annotation of these correlated genes. (**F**) The bubble plot shows the molecular function (MF) of the GO annotation of these correlated genes. (**G**) GSEA demonstrates the predicted upregulated (**left**) and downregulated (**right**) pathways in cancers with high expression of SLC19A1.

As multi-omics data support the prognostic value of SLC19A1, it was hypothesized SLC19A1 drives the downstream cascades to promote cancer progression. Hence, the interacted protein of SLC19A1 was obtained and visualized by STRING database according to previously published studies [74]. PLEKHA4, HNRNPL, CD164L2, LMBR1L, KRT26, KRAS, and NRAS proteins were previously identified to directly interact with the SLC19A1 encoding protein according to the STRING database (Figure 4A). Based on the transcriptomic data of TCGA pan-cancer cohorts, the expression of RRP1B, RRP1, MAZ, DUS1L, and HGH1 was identified to correlate positively with the expression of SLC19A1 in 40 types of cancers (Figure 4B). These SLC19A1-correlated genes in different cancers were therefore annotated. Regarding the KEGG pathway, these genes are enriched significantly in pyrimidine metabolism, purine metabolism, N-glycan biosynthesis, aminoacyl-tRNA biosynthesis, metabolic pathways, drug metabolism, fatty acid biosynthesis, ribosome biogenesis, proximal tubule bicarbonate reclamation, and ubiquitin-mediated proteolysis (Figure 4C). GO annotation was also performed on these genes (Figure 4D–F). In terms of biological process, they are enriched significantly in DNA metabolic process, ribonucleoprotein complex biogenesis, and ribosome biogenesis (Figure 4D). For cellular components, the nuclear part, nuclear lumen, and nuclear plasm are enriched, and the enriched molecular functions include nucleic acid binding and RNA binding (Figure 4E,F). GSEA analysis suggests the upregulation of SLC19A1 contributes to the activation of multiple oncogenic pathways, such as myc and DNA repair, and also to the suppression of some immune-related pathways, including TNF-alpha and inflammatory response (Figure 4G). These findings indicate the poor prognosis of patients with a high SLC19A1 expression may be due to the neoplastic progression induced by the downstream oncogenic activation and immune suppression.

### 3.5. SLC19A1 Is Involved in Tumor Immune Infiltration and Cytokine-Mediated Immune Regulation

**Figure 5:**
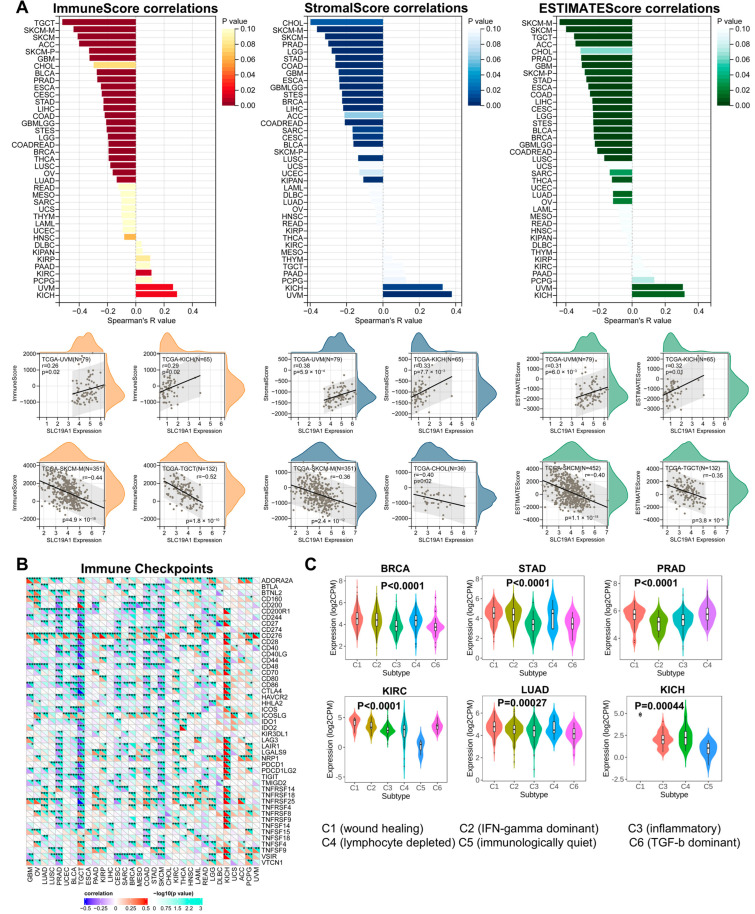
**SLC19A1 is correlated to immune infiltration and immune checkpoints.** (**A**) The bar charts show the R values of the correlations between the SLC19A1 expression and immune scores, stromal scores, and estimate scores, which were obtained from the ESTIMATE algorithm. The scatter plots below demonstrate the correlation between SLC19A1 expression and these scores in representative cancers. (**B**) The heatmap demonstrates the correlation between the transcriptomic expression of SLC19A1 and immune checkpoints. * *p* < 0.05, ** *p* < 0.01, and *** *p* < 0.001. (**C**) The violin plots demonstrate the SLC19A1 expression in different immune subtypes of multiple cancers.

In order to further study SLC19A1’s association with cancer immunity, a deconvolution algorism was applied to determine the immune infiltration based on the transcriptomic data of the TCGA pan-cancer cohorts. The immune infiltration level was quantified by an immune score, and its correlation with SLC19A1 was significantly negative in 19 types of analyzed cancers (n = 19/39), including TCGT, SKCM, ACC, GBM, BLCA, PRAD, ESCA, CESC, STAD, LIHC, COAD, LGG, STES, READ, BRCA, THCA, LUSC, OV, and LUAD (Figure 5A). In UVM and KICH, the correlation was significantly positive (Figure 5A). Regarding the infiltrated stroma cells and tumor purity, SLC19A1 was also widely associated with them in various tumors (Figure 5A). The correlation between SLC19A1 expression and immune checkpoints was also visualized. SLC19A1 was negatively correlated with the expression of most immune checkpoints in most cancers, except in KICH (Figure 5B). The different distribution of SLC19A1 expression among different immune subtypes, including wound healing (C1), IFN-gamma dominant (C2), inflammatory (C3), lymphocyte depleted (C4), immunological quiet (C5), and TGF-beta dominant (C6), was explored. In BRCA, STAD, PRAD, KIRC, LUAD, and KICH, the SLC19A1 was expressed significantly different among different immune subtypes (Figure 5C). In KIRC and KICH, the immunological quiet (C5) subtype demonstrated significantly low SLC19A1 expression (Figure 5C), suggesting that SLC19A1 may be associated with lower immune responses in these two tumors. In summary, these findings suggest that SLC19A1 is associated with cancer immunity and immune checkpoint defects in various cancers. Cancer immunity and immune checkpoint expression largely affect tumor prognosis, suggesting that the effect of SLC19A1 on the prognosis of different tumors may be achieved through cancer immunity.

In addition, we analyzed the association between SLC19A1 and chemokines, receptors, and major histocompatibility complex (MHC). As shown in the heatmap (Supplementary Figure S3), SLC19A1 was negatively correlated with most chemokines, receptors, and MHC in a pan-cancer context; however, SLC19A1 was broadly positively correlated with these cytokines in KICH and UVM. In addition, some specific chemokines (such as CXCL1, 2, 3), receptors (CXCR5 and CCR10), and MHC (TAP1, 2 and TAPBP) were also positively correlated with SLC19A1 in various tumors.

### 3.6. SLC19A1 Is Associated with the Absence of CD8-Positive T Cells and the Infiltration of M0 and M2 Macrophages

**Figure 6:**
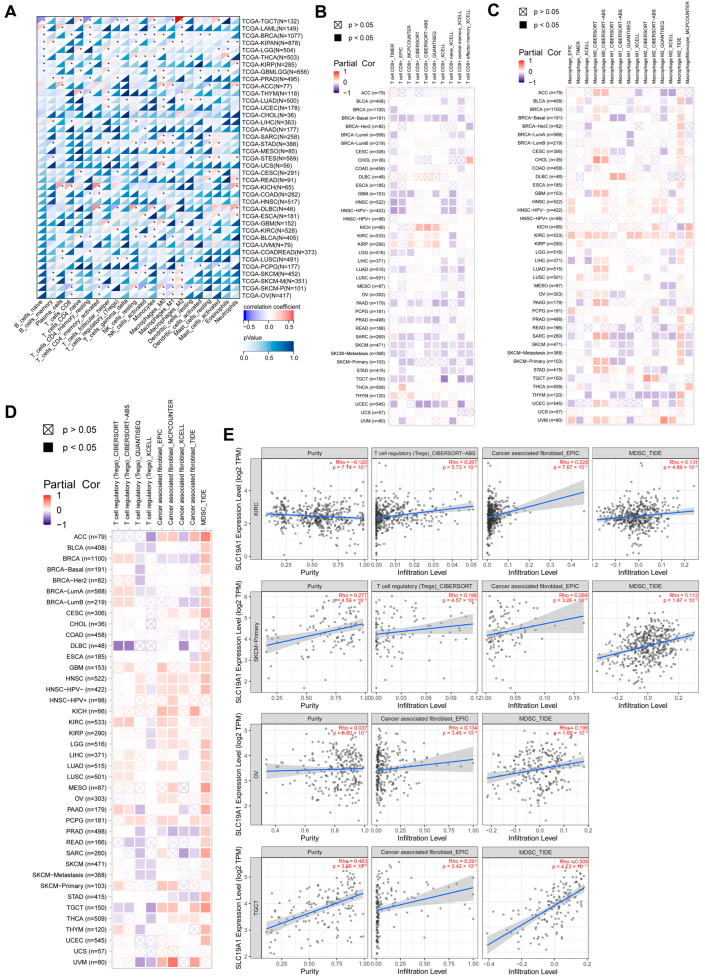
**SLC19A1 is associated with the absence of CD8-positive T cells and the infiltration of M0 and M2 macrophages.** (**A**) The heatmap demonstrates the correlation between the transcriptomic expression of SLC19A1 and the infiltrated immune cells. * *p* < 0.05. (**B**) The heatmap demonstrates the correlation between the transcriptomic expression of SLC19A1 and the infiltrated CD8-positive T cells. (**C**) The heatmap demonstrates the correlation between the transcriptomic expression of SLC19A1 and the infiltrated macrophages. (**D**) The heatmap demonstrates the correlation between the transcriptomic expression of SLC19A1 and the infiltrated T-regulatory cells and cancer-associated fibroblasts. (**E**) The scatterplots demonstrate the correlation between the transcriptomic expression of SLC19A1 and the infiltrated cells in specific cancers.

Considering its commonly negative association with immune infiltration, specific infiltrated immune cells were explored. The infiltration of B cells, T cells, NK cells, macrophages, etc., was assessed, and the correlation analysis with SLC19A1 expression suggested a lack of infiltrated anti-tumor immune cells is commonly seen in high SLC19A1 expression cancers (Figure 6A). Various deconvolution algorisms suggested SLC19A1 expression is negatively correlated with CD8-positive T cells in most of the cancers analyzed, except KICH, KIRC, KIRP, and THYM (Figure 6B). For non-activated M0 macrophages, the CIBERSORT algorithm showed that SLC19A1 is generally positively correlated with them (Figure 6C). Notably, inference based on the TIDE algorithm showed that SLC19A1 is associated with increased infiltration of M2 macrophages in most tumors (Figure 6C). Although no specific patterns of T-reg cells and cancer associated fibroblasts were identified, myeloid-derived suppressor cells (MDSCs) were found to be positively associated with SLC19A1 in majority of the cancer analyzed (Figure 6D). Specifically, SLC19A1 expression correlated positively with T-regs, cancer-associated fibroblasts, and MDSCs in KIRC and SKCM-primary (Figure 6E). In OV and TCGT, cancer-associated fibroblasts and MDSC also correlated positively with SLC19A1 (Figure 6E).

**Figure 7:**
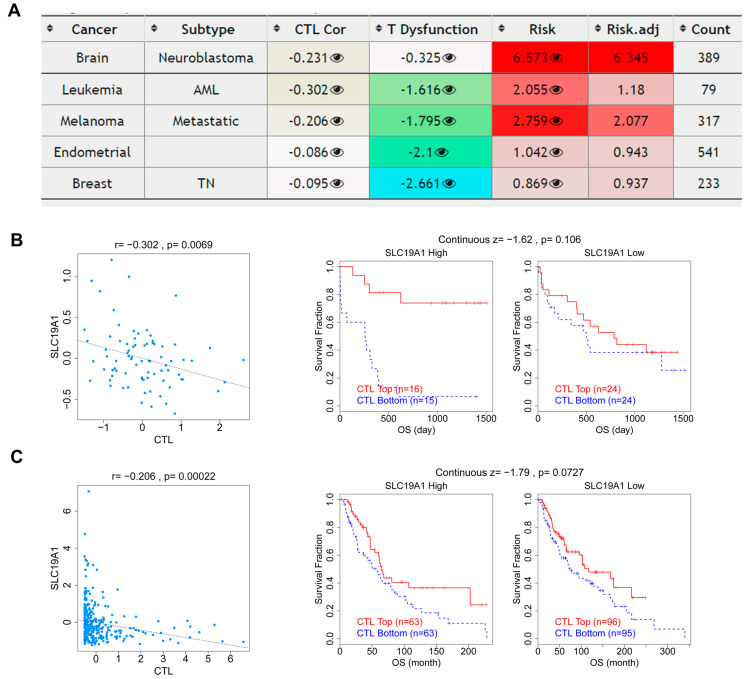
**The correlations between SLC19A1 expression and CTL, CTL dysfunction, and risks.** (**A**) The table presents the correlation between SLC19A1 expression, CTLs, and T-cell dysfunction. (**B**) The scatterplot (**left**) shows the correlation between the transcriptomic expression of SLC19A1 and the infiltration of CTLs in AML. The Kaplan–Meier curves show the survival of patients with high and low CTLs infiltration in patients with high (**middle**) and low SLC19A1 expression (**right**) in the AML cohort. (**C**) The scatterplot (**left**) shows the correlation between the transcriptomic expression of SLC19A1 and the infiltration of CTLs in metastatic melanoma. The Kaplan–Meier curves show the survival of patients with high and low CTLs infiltration in patients with high (**middle**) and low SLC19A1 expression (**right**) in the metastatic melanoma cohort.

Furthermore, the correlations among CTLs, T-cell dysfunction, survival, and SLC19A1 expression were overviewed, revealing significant associations in neuroblastoma, acute myeloid leukemia (AML), metastatic melanoma, uterine corpus endometrial carcinoma, and triple-negative BRCA (Figure 7A). In AML, the expression of SLC19A1 correlated negatively with CTLs. In addition, higher infiltration of CTLs was found to predict a better prognosis and serve as a prognostic factor in patient groups with high and low SLC19A1 expression (Figure 7B). Similar results were found in metastatic melanoma (Figure 7C). In summary, high SLC19A1 expression predicts reduced CD8-positive T cells and is associated with M0 and M2 macrophages.

### 3.7. The Protein Characterization of SLC19A1 Reveals Its Higher Expression in Certain Tumors and Correlates with M2 Macrophage Infiltration

**Figure 8:**
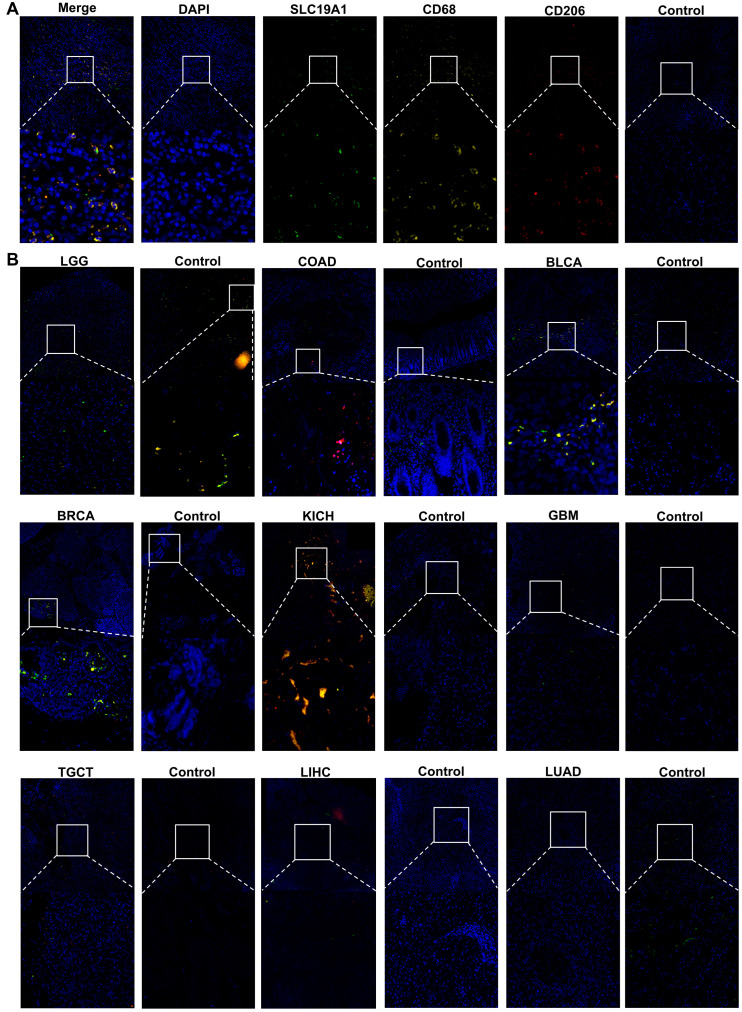
**Multiple fluorescence staining of SLC19A1 in pan-cancer tissue chips.** (**A**) The images show the immunofluorescence (IF) staining for DAPI (blue), SLC19A1 (green), CD68 (yellow), and CD163 (red) of STAD tissue and its adjacent normal tissue. A merged image and images of each channel are shown. Images at 20× magnification are shown at the top and enlarged images at the bottom. (**B**) The merged images for IF staining of the above markers of tumor and normal tissue are shown. The box in the figure represents the selected field of view, and the dotted line represents the enlargement of the selected field of view as shown below.

Given its association with immune infiltrations, immunofluorescence (IF) staining was performed in several tumors and paired normal samples to determine the spatial proteomic expression of SLC19A1 and infiltrated immune cells. Live cell nucleuses were marked by 4′,6-diamidino-2-phenylindole (DAPI) in the blue channel, while SLC19A1 protein was marked by its antibody in the green channel (Figure 8A). The CD68 antibody marks immune cells of the monocyte lineage in the yellow channel (Figure 8A). CD163 (red) and CD68-double-positive cells represent M2-type macrophages (Figure 8A). Subsequently, this antibody panel was applied to STAD, LGG, COAD, BLCA, BRCA, KICH, GBM, TGCT, LIHC, and LUAD and their paired normal tissue (Figure 8). Compared to the normal tissue, STAD was expressed relatively higher SLC19A1, while a higher infiltration of M2 macrophages was also found in the tumor tissue (Figure 8A, Supplementary Figure S4A). Similar trends were identified in BLCA and KICH compared to their paired normal tissue (Figure 8B, Supplementary Figure S4B,C). However, the opposite was found in LGG. The normal tissue expressed relatively more SLC19A1 proteins and infiltration of more monocytes and macrophages compared to LGG (Figure 8B, Supplementary Figure S4D). In BRCA, although tumor tissue partially expressed more SLC19A1, the single-CD68-positive cells indicated the majority of infiltrated immune cells were macrophages or monocytes instead of M2 macrophages (Figure 8B, Supplementary Figure S4E). In the rest of the samples, the protein expression of SLC19A1 and immune cells was not well identified (Figure 8B, Supplementary Figure S4F). These findings suggest that the proteomic expression of SLC19A1 is higher in certain tumors compared to their paired normal tissues and predicts a higher infiltration of M2 macrophages. The results in LGG suggest that the reverse may also be true and that larger cohorts are needed to fully understand the relationship between SLC19A1 and M2 macrophages.

### 3.8. Higher SLC19A1 Predicts Resistance to Chemotherapy and Immunotherapy in Several Cancers

**Figure 9:**
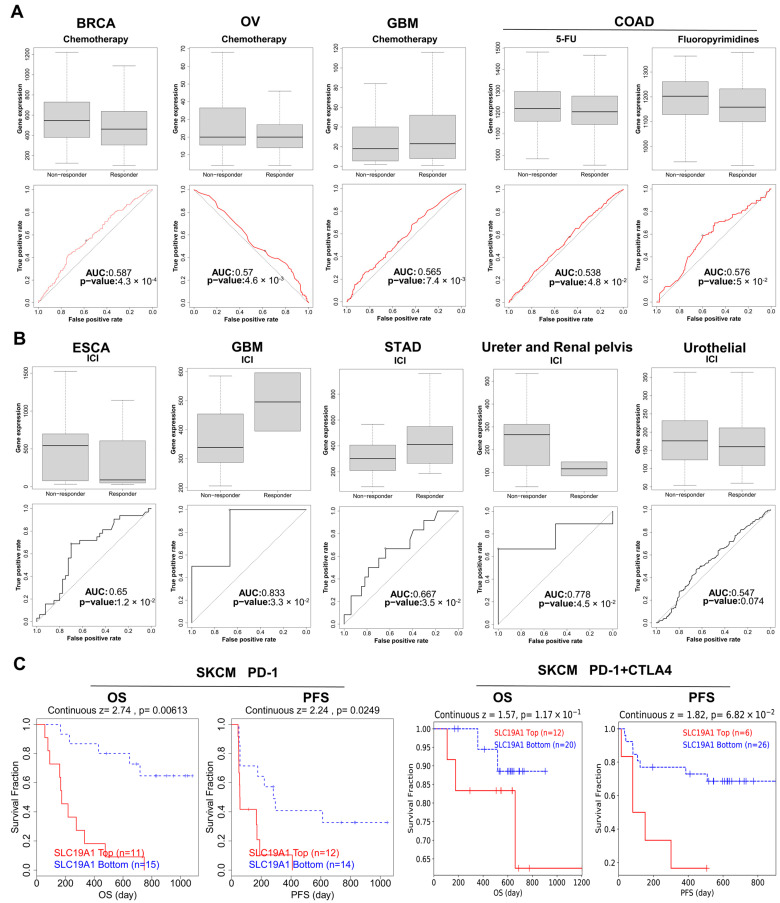
**SLC19A1 predicts therapeutic responses.** (**A**) The box plots demonstrate the SLC19A1 expression in non-responder and responder in cancer patients treated with chemotherapies. The Receiver Operating Characteristic (ROC) curves show the performance of the SLC19A1 in predicting the chemotherapeutic response. AUC, Area under the curve. (**B**) The box plots demonstrate the SLC19A1 expression in non-responder and responder in cancer patients treated with immune checkpoint inhibitors (ICI). The Receiver Operating Characteristic (ROC) curves show the performance of the SLC19A1 in predicting the ICI response. AUC, Area under the curve. (**C**) The Kaplan–Meier curves (**left**) show the OS and PFS of SKCM patients treated with PD1 with high and low SLC19A1 expression. The Kaplan–Meier curves (**right**) show the OS and PFS of SKCM patients treated with PD-1 and CTLA4 inhibitor with high and low SLC19A1 expression.

Considering its prognostic value in patient survival, it was hypothesized that SLC19A1 can also be employed to guide the treatment strategies. Therefore, the drug response of chemotherapies in different cancers and the SLC19A1 expression was obtained and compared. In the BRCA cohort compared to the no-response group, the SLC19A1 expression was significantly lower in the group with chemotherapeutic response (Figure 9A). In other words, elevated SLC19A1 expression is indicative of a lack of response to chemotherapy in BRCA. A comparable result was observed in the OV cohort (Figure 9A). In the COAD cohort, high expression of SLC19A1 predicted a poorer response to chemotherapies in both 5-FU- and fluoropyrimidines-treated patients (Figure 9A). However, the opposite was found in the GBM cohort (Figure 9A). For immune checkpoint inhibitor (ICI), a heterogenous response pattern was identified across different cancers (Figure 9B). Specifically, in ESCA, ureter and renal pelvis cancers, and urothelial carcinoma, higher SLC19A1 expression was associated with poorer response (Figure 9B). On the contrary, in GBM and STAD, higher SLC19A1 expression predicted a better response to ICI (Figure 9B). Interestingly, for SKCM patients treated with PD-1 or the combination of PD-1 and CTLA4 inhibitor, Kaplan–Meier curves suggest that patients with higher expression level of SLC19A1 have a significantly shorter lifespan compared to those with lower SLC19A1 expression (Figure 9C). Although a heterogeneous response profile to ICI was identified in some cancers, for classical chemotherapies and PD-1 treatment, SLC19A1 could be employed for risk stratification in specific cancers. In summary, among patients with BRCA, OV, and COAD undergoing chemotherapy, those with low SLC19A1 expression tend to have better survival benefits, indicating a more favorable prognosis. Conversely, in GBM patients undergoing chemotherapy, those with high SLC19A1 expression are more likely to reap survival benefits. Regarding ICI therapy, patients with low SLC19A1 expression in ESCA, ureter and renal pelvis cancers, and urothelial cancers are more likely to achieve survival benefits, whereas those with high SLC19A1 expression in GBM and STAD are more likely to benefit from the treatment.

## 4. Discussion

In addition to the well-defined function as a folate transporter, SLC19A1 was recently found to serve as a specific carrier that transport the extracellular cyclic dinucleotides inward [10,11]. Given the pivotal function of cyclic dinucleotides in cancer and infection immunity, it was hypothesized that SLC19A1 could serve as a key molecule for the management of cancers [81]. Hence, in order to initially overview the dysfunction of SLC19A1 in different cancers, a transcriptomic analysis of SLC19A1 expression was performed on a pan-cancer scale, showing that SLC19A1 was expressed significantly higher in most of cancers analyzed, while an opposite expression was found in a few cancers. The survival analysis suggests that higher transcriptomic expression of SLC19A1 is commonly associated with unfavorable prognosis. Simultaneously, genetic alterations and the encoding protein of SLC19A1 were also overviewed. Both of them were widely upregulated compared to normal tissues, as seen at the transcriptomic level. Integrated follow-up data also suggest that the genetic alterations of SLC19A1 predict a poorer survival. These results as well as the epigenetic analysis of SLC19A1 in different cancers confirm the hypothesis that SLC19A1 plays an important role in cancer biology. Although the mechanism behind this has not been well elucidated, one study determined the prognostic value of SLC19A1 in osteosarcoma, which is consistent with the findings above [82]. In addition, the circular RNA formation of SLC19A1 was identified to promote the prostate cancer progression via extracellular vesicles [83].

Given the broad dysfunction and mechanisms still to be elucidated, the associated molecules were explored, leading to the identification of the activation of downstream oncogenic cascade. They are broadly associated with mRNA synthesis and translation, which all indicate the abnormal activation of cellular progression. Some oncogenic pathways, such as myc and DNA damage repair, were found to be activated by these related genes, while several essential pathways for cancer-immunity were simultaneously found to be suppressed. These findings are consistent with recent studies showing that SLC19A1 acts not only as a folate transporter but also as a carrier for cyclic dinucleotides that can activate STING to induce broad anti-infective and anti-cancer immunity [81]. Given this, we evaluated the association between immune infiltration and SLC19A1. Interestingly, although higher SLC19A1 expression in most tumors is generally associated with an overall lack of immune infiltration and downregulation of immune checkpoints, specifically a lack of CD8 T cells, there was a significant increase in infiltration of M2 macrophages and MDSCs. This partially explains the poor prognosis of patients with higher SLC19A1 expression from an immune perspective. It also suggests that although SLC19A1 activates the immune response in tumors, it may recruit tumor-promoting cells, which deserves further exploration in future studies.

Given the essential function of SLC19A1 in the anti-neoplastic effect of anti-folate compounds including pemetrexed and methotrexate [4,37], its potential role in other anti-cancer therapeutics was explored. Interestingly, resistance of several chemotherapies was found to associated with a higher expression of SLC19A1. Although a heterogeneous response profile was identified in different cancers, higher SLC19A1 expression predicted a poorer response to immunotherapies in several cancers. In SKCM patients treated with PD-1, those with higher SLC19A1 expression were found to have a significantly poorer prognosis. Considering distinct tumor microenvironment pattern and neoplastic behavior across different cancers, novel parameters are required to facilitate the risk stratification by SLC19A1. However, the survival difference and mechanisms explored in this study all suggest that SLC19A1 could serve as a potential marker for cancer management and could also be used as a novel target for anti-cancer drug development. Methotrexate and sulfasalazine have served as classic SLC19A1 inhibitors and are commonly used to treat rheumatoid arthritis and inflammatory bowel disease [84,85,86]. These anti-folates also act as anti-metabolites to facilitate cancer treatment by inhibiting the import of folate, which is essential for cancer metabolism [87]. Recent evidence suggests that SLC19A1 is also responsible for the transport of cyclic dinucleotides [10,11], thus supporting the anti-cancer effect of its inhibition. Given the successful development of folate analogs to inhibit SLC19A1, a novel treatment strategy through the development of SLC19A1 inhibitors seems possible. However, dysfunction of SLC19A1 could lead to resistance to anti-folate treatments in cancers [87]. Therefore, novel therapeutics targeting folate transporters with higher efficacy and broader spectrum may contribute to cancer treatment. In particular, cyclic dinucleotide analogs have shown remarkable anti-tumor efficacy in mouse models, leading to two phase I clinical trials with these cyclic dinucleotide analogs [11,29]. Given the recent evidence that SLC19A1 serves as a cyclic dinucleotide transporter [10,11], the combination of therapeutics targeting SLC19A1 and cyclic dinucleotide analogs may be a novel direction for cancer treatment.

This study offers a comprehensive pan-cancer analysis of SLC19A1, identifying it as an unfavorable prognostic marker across multiple cancer types. The integration of multi-omics data, including transcriptomic, proteomic, and genomic analyses, provides a robust framework for understanding the diverse roles of SLC19A1 in cancer biology. Furthermore, the exploration of its association with immune infiltration, particularly the correlation with M2 macrophages, adds valuable insights into the tumor microenvironment and its implications for immunotherapy. Despite these strengths, several limitations should be noted. First, while the correlations identified in this study are significant, the results rely on computational predictions, and the underlying mechanisms by which SLC19A1 influences tumor progression and immune modulation require further investigation. Second, the prognostic value of SLC19A1 may be affected by confounding factors. Additionally, the observed heterogeneity in immune and therapeutic responses related to SLC19A1 expression suggests a need for further exploration to understand the factors contributing to this variability. Lastly, future studies should aim to conduct functional validation experiments to elucidate the specific biological roles of SLC19A1 in various cancer contexts.

The translational implications of this study are twofold. First, SLC19A1 expression could serve as a clinical biomarker to stratify patients for personalized treatment strategies. For instance, elevated SLC19A1 levels in tumors such as BRCA, SKCM, and COAD may predict resistance to conventional chemotherapies and immunotherapies, enabling clinicians to prioritize alternative regimens for high-risk patients. Second, targeting SLC19A1 directly could disrupt its dual role in folate metabolism and immune suppression. Inhibitors of SLC19A1 might synergize with existing therapies to counteract chemoresistance or enhance anti-tumor immunity by reducing M2 macrophage recruitment and restoring CD8+ T-cell activity. Future studies should focus on validating these hypotheses in preclinical models and exploring SLC19A1-targeted compounds in early-phase clinical trials. Such efforts could translate our mechanistic insights into novel therapeutic interventions, ultimately improving outcomes for patients with aggressive or treatment-resistant cancers.

In conclusion, our comprehensive pan-cancer analysis of SLC19A1 underscores its potential as a significant prognostic biomarker across various malignancies. SLC19A1 appears to play a crucial role in tumor progression by facilitating folate transport and influencing immune microenvironments. Our findings suggest that SLC19A1 is linked to genomic instability and may contribute to the maintenance of cancer stemness and chemoresistance in neoplastic cells. Importantly, we established SLC19A1 as a marker for M2 macrophage infiltration, indicating its involvement in immune evasion mechanisms, including the suppression of CD8-positive T cells. These insights highlight the multifaceted roles of SLC19A1 in cancer biology and point to its potential as a therapeutic target. Given the implications of SLC19A1 in immune modulation and treatment resistance, further exploration of SLC19A1-targeted therapies by integrating clinical samples, molecular biology experiments, and prospective cohorts could lead to improved outcomes for patients facing aggressive cancers resistant to conventional treatments.

## Figures and Tables

**Table 1:** Abbreviations of cancer types.

Cancer Type	Definition
AML	Acute myeloid leukemia
ACC	Adrenocortical carcinoma
BLCA	Bladder urothelial carcinoma
LGG	Brain lower-grade glioma
BRCA	Breast invasive carcinoma
CESC	Cervical squamous cell carcinoma and endocervical adenocarcinoma
CHOL	Cholangiocarcinoma
COAD	Colon adenocarcinoma
ESCA	Esophageal carcinoma
GBM	Glioblastoma multiforme
HNSC	Head and neck squamous cell carcinoma
KICH	Kidney chromophobe
KIRC	Kidney renal clear cell carcinoma
KIRP	Kidney renal papillary cell carcinoma
LIHC	Liver hepatocellular carcinoma
LUAD	Lung adenocarcinoma
LUSC	Lung squamous cell carcinoma
DLBC	Lymphoid neoplasm diffuse large B-cell lymphoma
MESO	Mesothelioma
OV	Ovarian serous cystadenocarcinoma
PAAD	Pancreatic adenocarcinoma
PCPG	Pheochromocytoma and paraganglioma
PRCA	Prostate carcinoma
PRAD	Prostate adenocarcinoma
READ	Rectum adenocarcinoma
SARC	Sarcoma
SKCM	Skin cutaneous melanoma
STAD	Stomach adenocarcinoma
TGCT	Testicular germ cell tumors
THYM	Thymoma
THCA	Thyroid carcinoma
UCA	Uterine carcinosarcoma
UCEC	Uterine corpus endometrial carcinoma
UVM	Uveal melanoma

## Data Availability

The public datasets involved in this study are described and can be found in the corresponding method sections. Further information can be requested from the corresponding author.

## Supplementary Materials

The following supporting information can be downloaded at: https://www.mdpi.com/article/10.3390/biomedicines13030571/s1, Figure S1: Univariate Cox analysis of SLC19A1 in OS (A), DSS (B) and PFI (C); Figure S2: Expression and prognostic analysis of SLC19A1 in the internal glioma cohort; Figure S3: The association between SLC19A1 and chemokines (A), receptors (B), and MHC (C); Figure S4: Quantitative analysis of immunofluorescence staining.

## Abbreviations

The following abbreviations are used in this manuscript:

SLC19A1	Solute carrier family 19 member 1
STING	Stimulator of interferon genes
CPTAC	Clinical Proteomic Tumor Analysis Consortium
ICPC	International Cancer Proteogenome Consortium
OS	Overall survival
DSS	Disease-specific survival
PFI	Progression-free interval
CNV	Copy number variation
TIDE	Tumor immune dysfunction and exclusion
CNA	Copy number alteration
TMB	Tumor mutational burden
MSI	Microsatellite instability
HRD	Homologous recombination deficiency
DMPsi	Differentially methylated probes-based stemness index
CTL	Cytotoxic T lymphocytes
TCGA	The Cancer Genome Atlas
GEO	Gene Expression Omnibus
GTEx	Genotype-Tissue Expression
m1A	N1-methyladenosine
m5C	N1-methyladenosine
m6A	N6-methyladenosine
GSEA	Gene Set Enrichment Analysis
AML	Acute myeloid leukemia
ACC	Adrenocortical carcinoma
BLCA	Bladder urothelial carcinoma
LGG	Brain lower-grade glioma
BRCA	Breast invasive carcinoma
CESC	Cervical squamous cell carcinoma and endocervical adenocarcinoma
CHOL	Cholangiocarcinoma
COAD	Colon adenocarcinoma
ESCA	Esophageal carcinoma
GBM	Glioblastoma multiforme
HNSC	Head and neck squamous cell carcinoma
KICH	Kidney chromophobe
KIRC	Kidney renal clear cell carcinoma
KIRP	Kidney renal papillary cell carcinoma
LIHC	Liver hepatocellular carcinoma
LUAD	Lung adenocarcinoma
LUSC	Lung squamous cell carcinoma
DLBC	Lymphoid neoplasm diffuse large B-cell lymphoma
MESO	Mesothelioma
OV	Ovarian serous cystadenocarcinoma
PAAD	Pancreatic adenocarcinoma
PCPG	Pheochromocytoma and paraganglioma
PRCA	Prostate carcinoma
PRAD	Prostate adenocarcinoma
READ	Rectum adenocarcinoma
SARC	Sarcoma
SKCM	Skin cutaneous melanoma
STAD	Stomach adenocarcinoma
TGCT	Testicular germ cell tumors
THYM	Thymoma
THCA	Thyroid carcinoma
UCA	Uterine carcinosarcoma
UCEC	Uterine corpus endometrial carcinoma
UVM	Uveal melanoma
