# Supplementary material for: Pan-Cancer Characterization Identifies SLC19A1 as an Unfavorable Prognostic Marker and Associates It with Tumor Infiltration Features
Source: Biomedicines. 2025 Feb 25;13(3):571. doi: 10.3390/biomedicines13030571 (PMC11940280; doi:10.3390/biomedicines13030571)
Supplement: Supplementary file 1 [file biomedicines-13-00571-s001.zip › biomedicines-3453580-supplementary.pdf]

Supplementary Figures

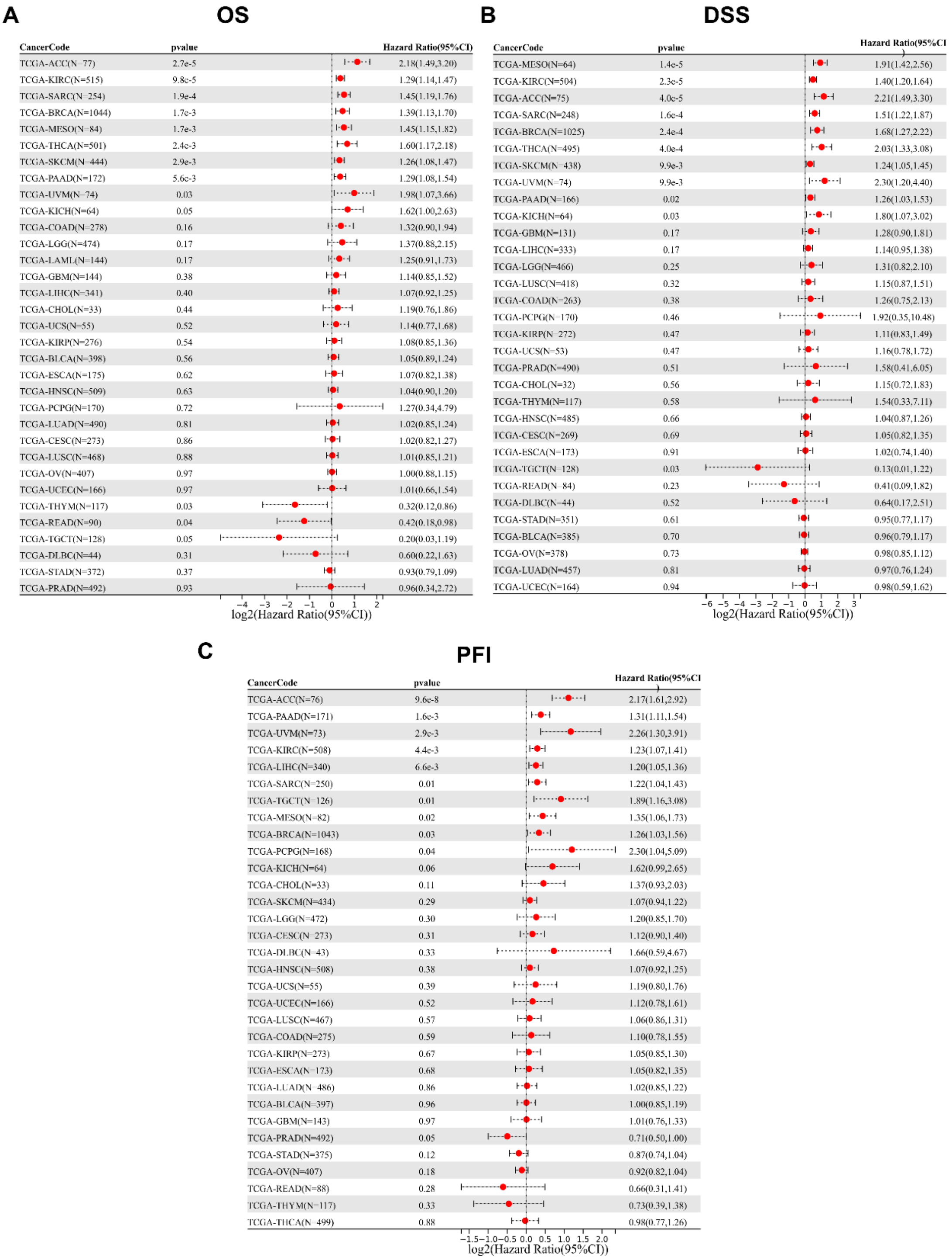

Figure S1. Univariate Cox analysis of SLC19A1 in OS (A), DSS (B) and PFI (C).

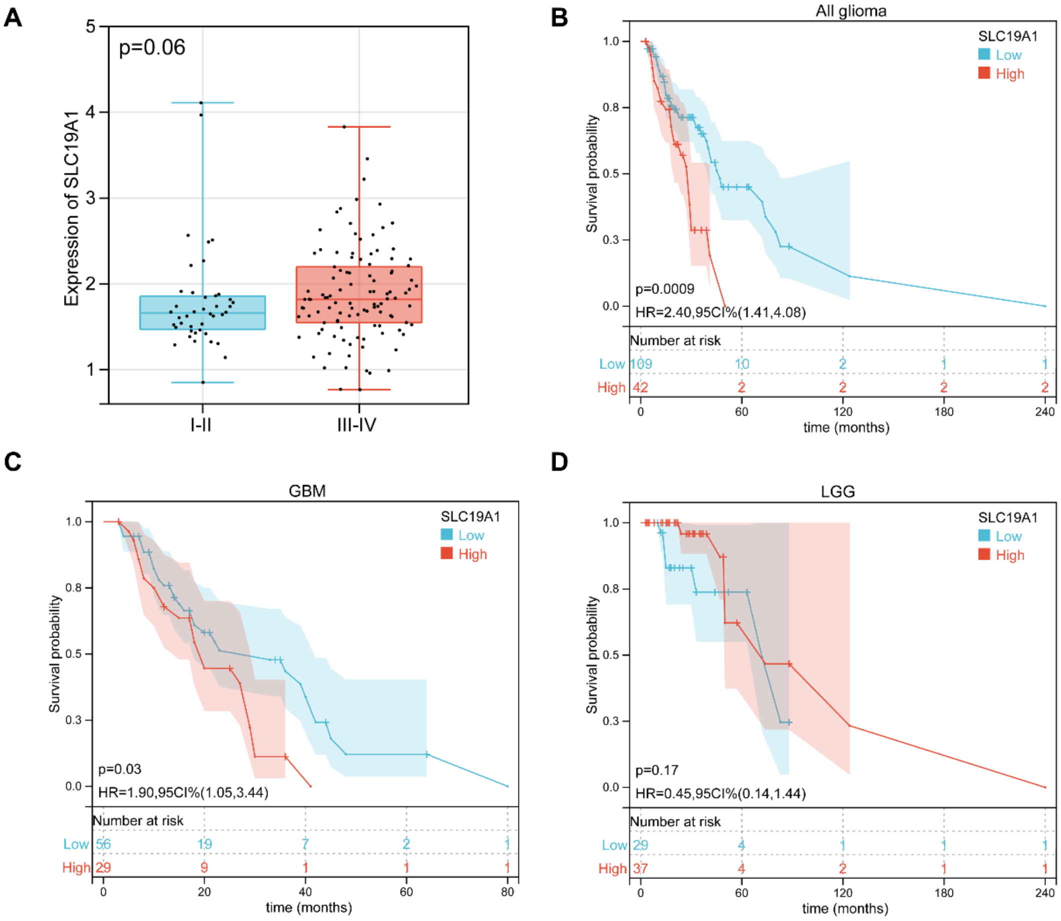

Figure S2. Expression and prognostic analysis of SLC19A1 in the internal glioma cohort. (A) Differential expression of SLC19A1 between grade I-II gliomas and grade III-IV gliomas. **(B-D)** Kaplan-Meier curves of all glioma patients **(B)**, GBM patients **(C)** and LGG patients **(D)** with high and low expression level of SLC19A1.

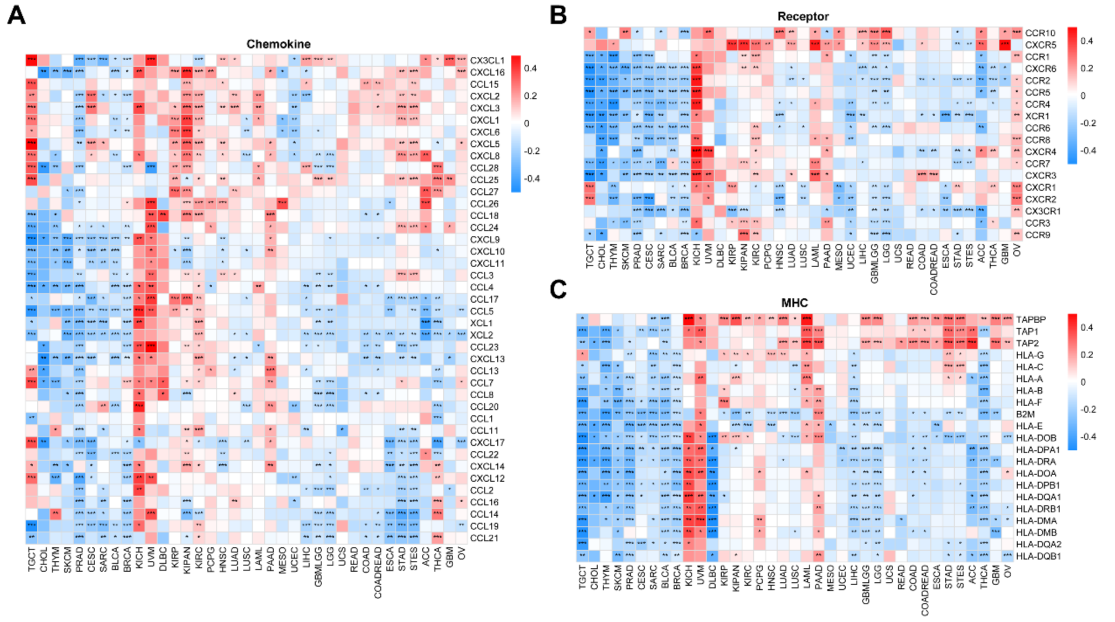

Figure S3. The association between SLC19A1 and chemokines (A), receptors (B), and MHC (C).

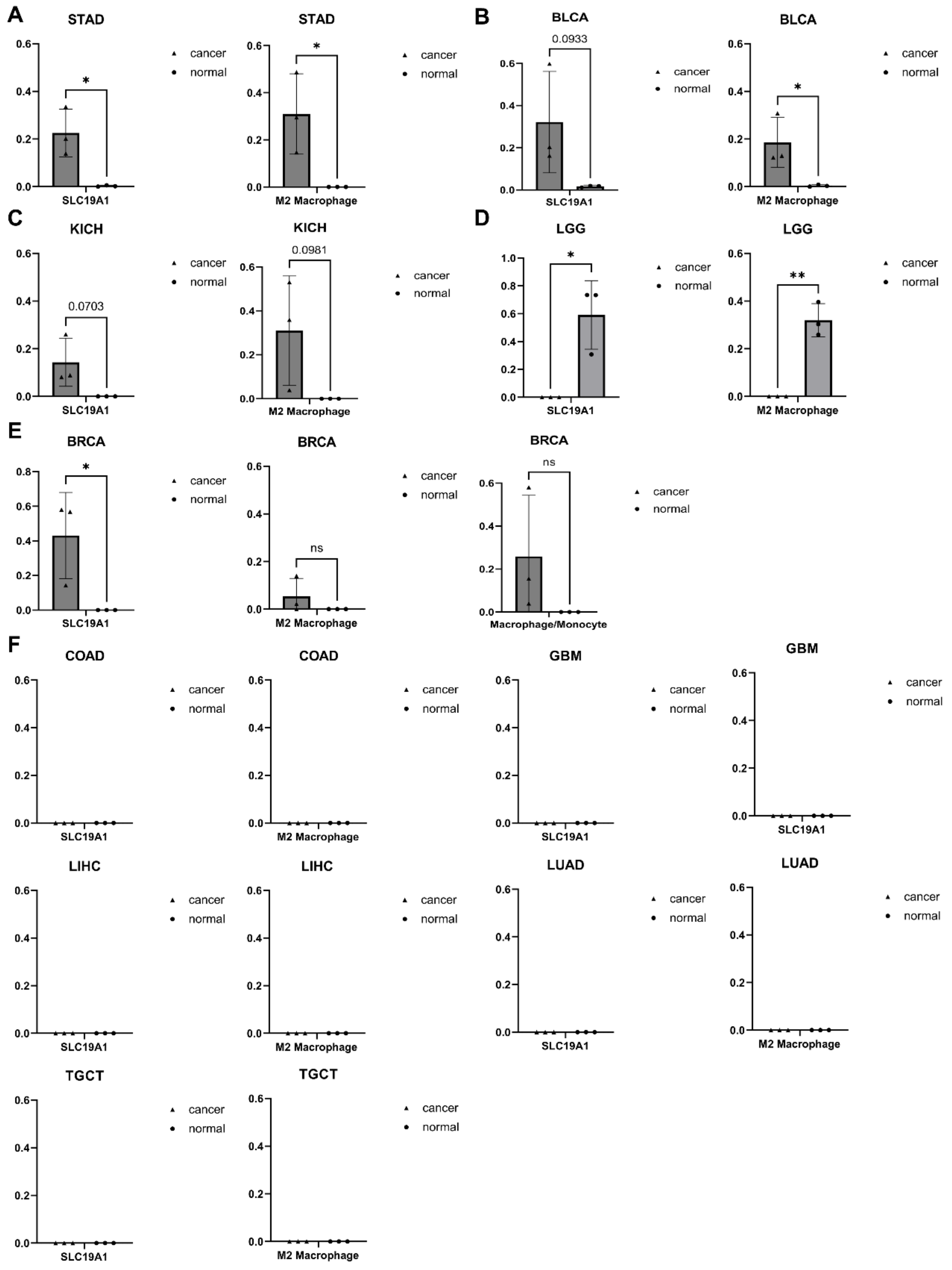

Figure S4. **Quantitative analysis of immunofluorescence staining.**
